# Human iPSC differentiation to retinal organoids in response to IGF1 and BMP4 activation is line‐ and method‐dependent

**DOI:** 10.1002/stem.3116

**Published:** 2019-12-30

**Authors:** Valeria Chichagova, Gerrit Hilgen, Ali Ghareeb, Maria Georgiou, Madeleine Carter, Evelyne Sernagor, Majlinda Lako, Lyle Armstrong

**Affiliations:** ^1^ Newcells Biotech Ltd Newcastle upon Tyne UK; ^2^ Institute of Neuroscience Newcastle University Newcastle upon Tyne UK; ^3^ Institute of Genetic Medicine Newcastle University Newcastle upon Tyne UK

**Keywords:** differentiation, induced pluripotent stem cells, organoids, retina

## Abstract

Induced pluripotent stem cell (iPSC)‐derived retinal organoids provide a platform to study human retinogenesis, disease modeling, and compound screening. Although retinal organoids may represent tissue structures with greater physiological relevance to the in vivo human retina, their generation is not without limitations. Various protocols have been developed to enable development of organoids with all major retinal cell types; however, variability across iPSC lines is often reported. Modulating signaling pathways important for eye formation, such as those involving bone morphogenetic protein 4 (BMP4) and insulin‐like growth factor 1 (IGF1), is a common approach used for the generation of retinal tissue in vitro. We used three human iPSC lines to generate retinal organoids by activating either BMP4 or IGF1 signaling and assessed differentiation efficiency by monitoring morphological changes, gene and protein expression, and function. Our results showed that the ability of iPSC to give rise to retinal organoids in response to IGF1 and BMP4 activation was line‐ and method‐dependent. This demonstrates that careful consideration is needed when choosing a differentiation approach, which would also depend on overall project aims.

## Graphical abstract

Three human induced pluripotent stem cell (iPSC) lines were differentiated to retinal organoids using differentiation protocols activating either bone morphogenetic protein 4 or insulin‐like growth factor 1 signaling pathways. All cell lines were able to generate retinal organoids. However, the two differentiation approaches produced bias toward certain retinal cell types within the organoids, which was iPSC line‐ and method‐dependent.

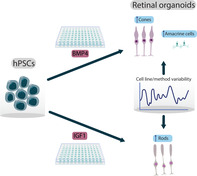

**Significance statement**

Retinal organoids were derived from three human induced pluripotent stem cell (iPSC) lines using two different differentiation approaches involving either bone morphogenetic protein 4 or insulin‐like growth factor 1 signaling pathways. Retinal organoids were generated using both methods; however, the two different approaches produced bias toward certain retinal cell types. The results of this study suggest that careful consideration is needed when choosing a differentiation protocol and that overall efficiency to generate retinal organoids would depend on the signaling pathways that are modulated.

## 1 INTRODUCTION

The development of in vitro retinal models has been driven by a lack of adequate animal models that recapitulate the structure and function of the human retina. Induced pluripotent stem cell (IPSC)‐derived retinal organoids have been shown to have a wide range of applications, including the study of human retinogenesis,^1^, ^2^, ^3^ disease modeling,^4^, ^5^ drug discovery,^6^, ^7^ and cell therapy.^8^, ^9^, ^10^ Numerous protocols have been developed for the generation of retinal organoids that follow basic developmental principles of forebrain development and eye formation. Despite the ability of these protocols to give rise to laminated retinal organoids, variability in the propensity of iPSCs to give rise to various retinal cell types is often reported. Several groups including ours have reported variable laminar organization between samples differentiated from the same iPSC lines and the presence of non‐neural cell types alongside the retinal structures.^7^, ^11^, ^12^

Retinal development in vivo is controlled by a diverse set of signaling pathways and complex interactions between embryological tissues which affect the identity of the resultant cell population. Forebrain development is orchestrated by a fine balance between a number of signaling pathways, including bone morphogenetic protein 4 (BMP4) and insulin‐like growth factor 1 (IGF1).^13^, ^14^ Long‐term maturation of retinal cells for an extended period of time is enhanced by retinoic acid (RA), taurine, and triiodothyronine (T3).^1^, ^15^, ^16^ We used differentiation protocols that followed these principles in order to compare retinal differentiation efficiency of multiple iPSC lines across differentiation protocols that rely on activation of these pathways.^7^, ^11^, ^17^, ^18^

## 2 RESULTS

**Figure 1:**
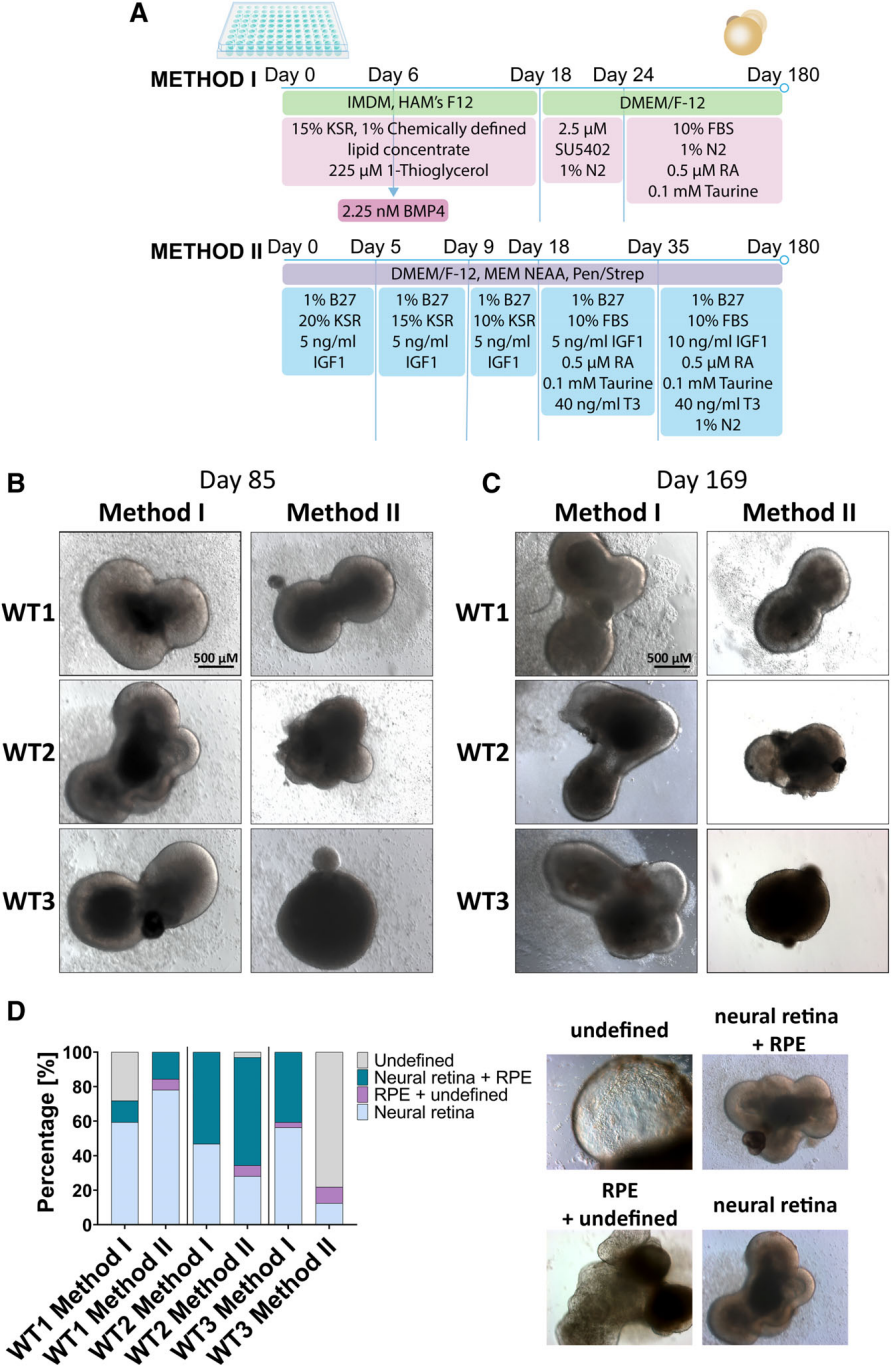
Generation of retinal organoids by modulating bone morphogenetic protein 4 and insulin‐like growth factor 1 signaling pathways. A, A schematic representation of the two differentiation methods. B and C, Bright‐field images showing development of retinal organoids at days 85 and 169 of differentiation. D, Representative examples and quantification of retinal and nonretinal tissues showing variability across methods and cell lines. n = 32 for each time point

To investigate the reproducibility of retinal organoid differentiation protocols across multiple iPSC lines, we differentiated three iPSC lines (WT1, WT2, and WT3.^7^) from unaffected subjects using two differentiation protocols, designated as Method I and Method II (details are shown in Figure 1A). Key morphological features of developing retina in vitro include the appearance of optical vesicles (OVs) containing phase‐bright neuroepithelium on the apical side, with some OVs also containing retinal pigment epithelium (RPE). Morphological observations at days 85 and 169 of differentiation showed that WT1 and WT2 organoids differentiated using both methods contained OV‐like structures and in some cases presumptive RPE cells identified by their pigmented appearance. WT3 organoids were comparable with WT1 and WT2 in Method I but responded poorly to Method II (Figure 1B,C). The capacity to give rise to neural retina with and without RPE differed across the lines and protocols (Figure 1D). WT1 and WT2 produced more RPE with Method II, WT3 responded better to Method I overall, and the number of undefined structures was cell line‐ and method‐dependent.

We performed quantitative PCR analysis to assess whether there was any difference in the expression of genes associated with various retinal cell types (photoreceptor precursors—cone‐rod homeobox [*CRX*]; photoreceptors and photoreceptor precursors—recoverin [*RCVRN*]; rods—neural retina leucine zipper [*NRL*], rhodopsin [*RHO*]; cones—short‐wave‐sensitive opsin‐1 [*OPN1SW*], medium‐wave‐sensitive opsin‐1 [*OPN1MW*], long‐wave‐sensitive opsin‐1 [*OPN1LW*]; horizontal cells—prospero‐related homeobox 1 [*PROX1*]; Müller glia and RPE—retinaldehyde‐binding protein [*RLBP*]; amacrine cells—activating enhancer‐binding protein 2‐alpha [*AP2α*]; retinal ganglion cells [RGCs]—atonal bHLH transcription factor 7 [*MATH5*], RNA binding protein, mRNA processing factor [*RBPMS*]; RPE—retinoid isomerohydrolase RPE65 [*RPE65*]) across differentiation protocols at 180 days of differentiation (Figure S1). Expression of *CRX* and *RHO* was significantly higher in WT1 organoids differentiated with Method II comparing to Method I; similarly, differentiating WT2 with Method II resulted in significantly higher expression of *NRL* comparing to organoids differentiated with Method I. These results are unsurprising since Method II uses T3, which is known to encourage rod development, which is reflected in upregulation of *NRL* and *RHO*.^19^ The largest difference between Methods I and II on differentiation outcome was observed in WT3 cells. Method I resulted in significant upregulation of all genes tested apart from *RBPMS* and *RPE65*, which corroborates the morphological observations reported in Figure 1D.

**Figure 2:**
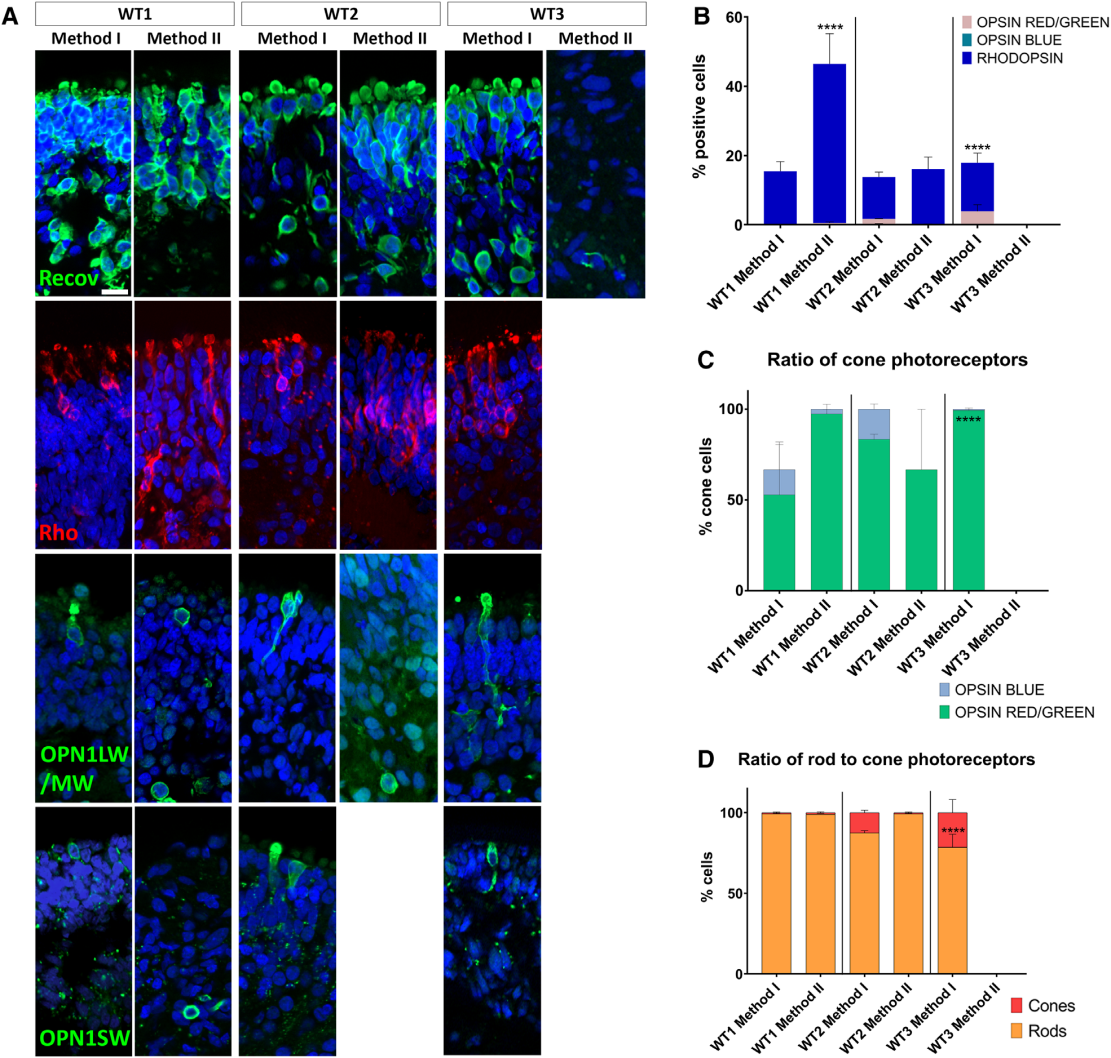
Development of photoreceptors. A, The expression of RECOVERIN, RHODOPSIN, OPSIN R/G, and OPSIN B indicated the presence of rod and cone photoreceptors. B, Quantification of proteins associated with different types of photoreceptors showed that both methods gave rise to rods and cones, apart from WT3 cells which were more responsive to Method I. C, Ratio of cone photoreceptors was in line with reported values (5‐8:1) in WT1 and WT2 Method I organoids. D, Organoids from all differentiations, apart from WT3 Method II, which failed to give rise to mature photoreceptors, contained a higher proportion of rods than cones. OPSIN B, opsin blue; OPSIN R/G, opsin red/green; Recov, recoverin; Rho, rhodopsin. Data are shown as mean ± SEM. Organoids used for these experiments were at day 180 of differentiation. Scale bar = 10 μm. ****P < .0001 for panels B‐D

Gene expression analyses were followed by immunofluorescence microscopy examining the presence of photoreceptors marked by the appearance of cells positive for RECOVERIN, RHODOPSIN, OPSIN RED/GREEN, and OPSIN BLUE (Figure 2A). Method I resulted in the development of photoreceptors in organoids from all iPSC lines, whereas Method II gave rise to photoreceptors in both WT1 and WT2 lines, with only negligible presence of RECOVERIN‐positive cells in the inner layers in WT3 organoids. There was significantly higher number of RHODOPSIN‐positive cells in WT1 organoids differentiated with Method II (Figure 2B); WT2 organoids differentiated with the same method showed the presence of RECOVERIN‐ and RHODOPSIN‐ positive cells and OPSIN RED/GREEN positive cells could also be observed; however, protein localization to the outer segments was not always apparent. The reported M/L‐ to S‐cone (red/green to blue) ratios range between 5 and 8 to 1.^1^, ^12^ We observed similar proportions in WT1 and WT2 Method I‐derived organoids (Figure 2C). Organoids from all differentiations, apart from WT3 Method II, which failed to give rise to mature photoreceptors, all contained a higher proportion of rods (Figure 2D).

**Figure 3:**
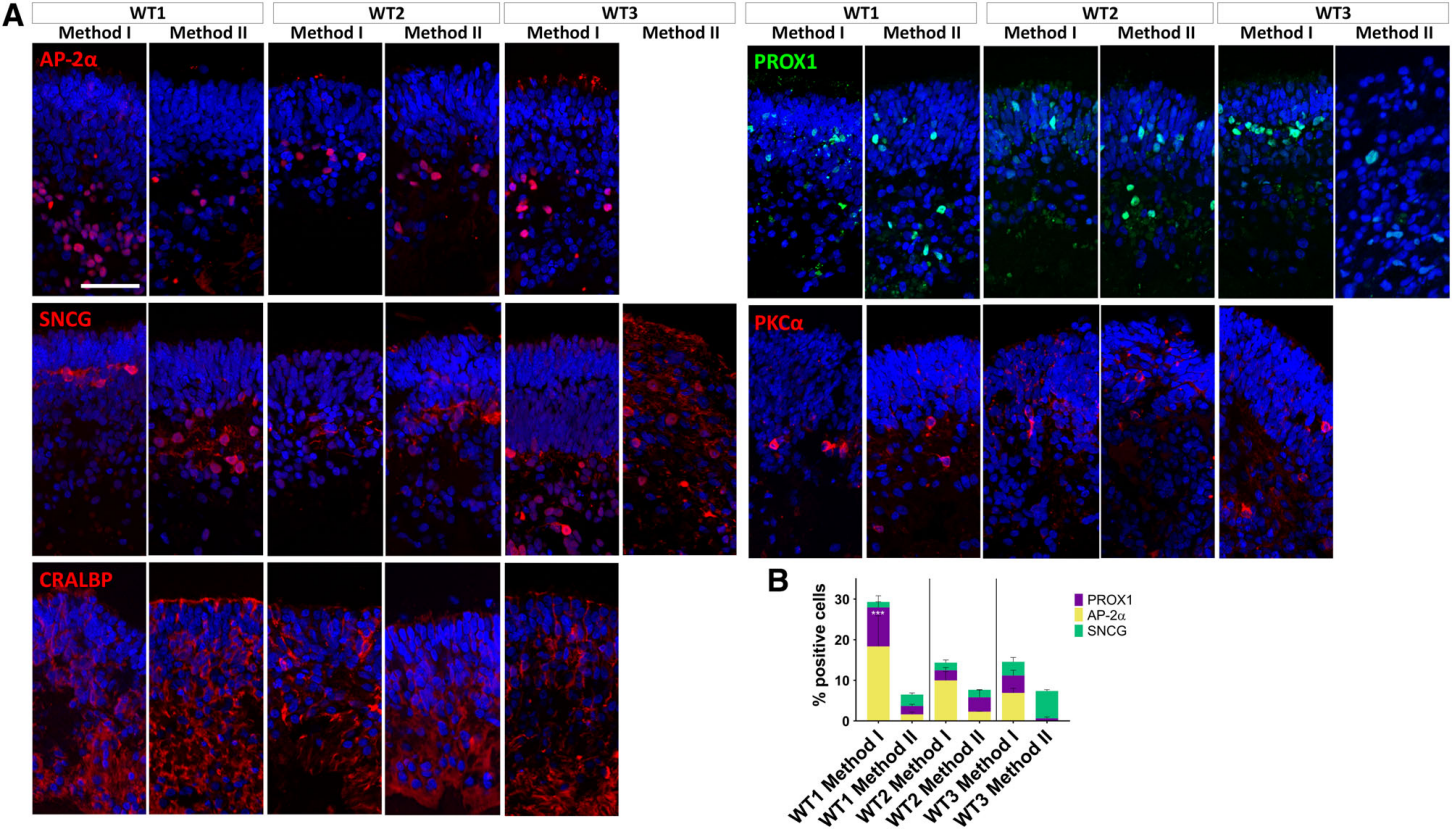
Development of retinal ganglion cells (RGCs), Müller glia, and differentiating neurons of the inner nuclear retinal layer (horizontal, amacrine, and bipolar cells). A, WT1 and WT2 cells differentiated with both methods and WT3 cells differentiated with Method I gave rise to cells positive for AP‐2α, SNCG, CRALBP, PROX1, and PKCα. WT3 cells differentiated with Method II only gave rise to SNCG and PROX1 positive cells. B, Method I gave rise to more amacrine cells (AP‐2α) in all cell lines (***P < .001 for WT1 Method I), apart from WT3 Method II, which did not have any cells positive for this marker. The number of RGCs (SNCG) was comparable across the methods. Müller glia (CRALBP) spanned across the retinal layers in all conditions, apart from WT3 Method II. The number of PROX1 positive cells was comparable across the conditions, apart from WT3 Method II, with only a small proportion of cells expressing it. Data are shown as mean ± SEM. Organoids used for these experiments were at day 180 of differentiation. Scale bar = 50 μm

In addition to photoreceptors, we also looked at the presence and distribution of amacrine (AP‐2α), RGCs (SNCG), Müller glia (CRALBP), and differentiating neurons of the inner nuclear retinal layer (horizontal/amacrine cells; PROX1; Figure 3A). Method I gave rise to more amacrine cells in all iPSC lines, with a significantly higher number of AP‐2α‐positive cells in WT1 Method I, comparing to Method II (Figure 3B). No amacrine cells were found in WT3 Method II. The number of RGCs was comparable across the methods with WT3 differentiated with Method II having a tendency to produce more cells positive for SNCG. Müller glia spanned across the retinal layers in all conditions, apart from WT3 Method II. The number of PROX1‐positive cells was comparable across the conditions, apart from WT3 Method II, with only a small proportion of cells expressing it. Overall, WT3 cells did not respond well to Method II, which is reflected by gene and protein expression data (Figures 2 and 3 and Figure S1).

**Figure 4:**
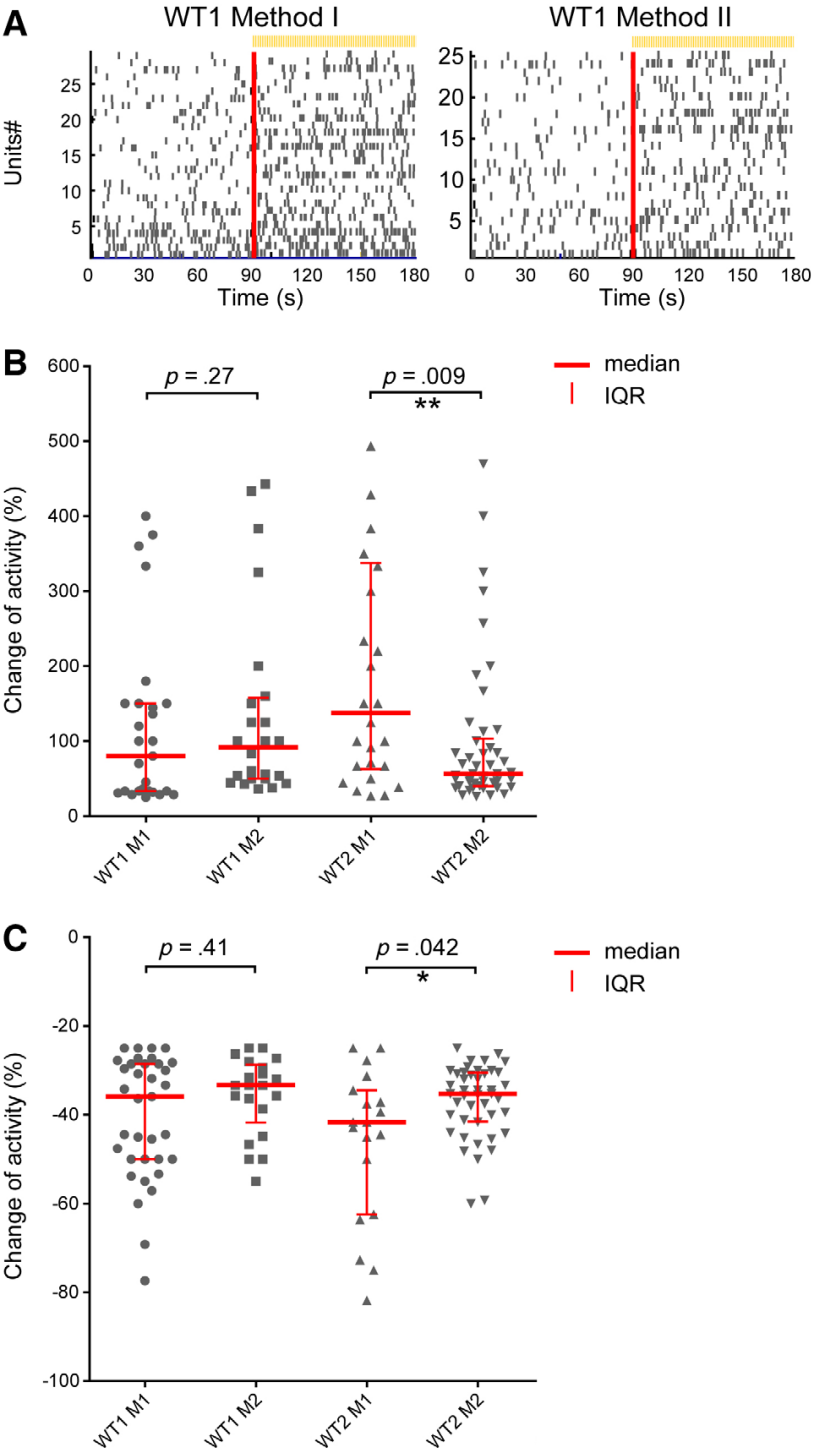
Light‐driven spiking activity recorded from presumed ON‐Centre retinal ganglion cells (RGCs) and OFF‐Centre RGCs. A, In the raster plot, each small vertical bar indicates the time stamp of a spike, where each row represents a different RGC. The left half illustrates the activity before stimulus onset and, separated by the red line, the right half the activity when exposed to light. B, The change of firing (COF) percentage values from presumed ON‐Centre RGCs are scatter plotted for Method I and Method II of WT1‐2 lines. The median is indicated as a red horizontal line and the interquartile range as a vertical line. Each symbol represents one RGC that showed more than 25% increased spiking after light onset. C, The COF percentage values from presumed OFF‐Centre RGCs are scatter plotted in the same way as described above. We defined outliers as values which exceed three times the standard deviation (three‐sigma rule). A total of five to six organoids were recorded from for each condition. Number of RGCs found to show increased firing in (B) WT1 (M1 = 27 out of 389 RGCs [∼6.9%]; M2 = 24/282 [8.5%]), WT2 (M1 = 26/317 [8.2%]; M2 = 46/496 [9.3%]). Number of RGCs found to show decreased firing in (C) WT1 (M1 = 35 of 389 RGCs [∼9%]; M2 = 21/282 [7.4%]), WT2 (M1 = 19/317 [6%]; M2 = 44/496 [8.8%])

As a final test, we compared the functionality in these organoids by quantifying their ability to respond to light. We have recently shown that retinal organoids can respond to light, similar to the earliest light responses in mice at day 150 of differentiation.^7^ Accordingly, we were able to record light‐driven spikes from retinal organoids from WT1 and WT2 iPSCs. Based on gene expression and immunofluorescence data (Figure S1, Figures 2A and 3A). WT1 and WT2 produced retinal cell types when differentiated with both methods, whereas WT3 responded poorly to Method II; therefore, we omitted WT3 from the analysis. RGCs were considered responsive if they showed at least 25% increase or decrease in spiking activity during a 90‐second time window after the onset of a white light pulse (200 milliseconds, 217 μW/cm^2^; irradiance, 1 Hz, duration 5 minutes) compared with the spiking rate before the light was turned on. Furthermore, we used sustained broad blue light stimulation (2 minutes, 217 μW/cm^2^ irradiance) to evoke responses from potential intrinsically photosensitive RGCs (ipRGCs). Photoreceptor‐driven responses are transient, whereas ipRGCs light responses are sustained and have a delayed onset. Hence, we classified RGCs as potential ipRGCs if they still exhibited significantly higher firing rates at least 30 seconds after the onset of the sustained blue stimulus. More importantly, all other RGCs, which showed relatively transient responses during sustained blue light, were classified as potentially photoreceptor‐driven and further analyzed in this study. The percentage of potential ipRGCs from the pool of all light‐driven RGCs for WT1 was 56.5% (Method I) vs 13.3% (Method II). For WT2, the ratio was 57.8%/60%. Overall, the photoreceptor‐driven RGCs in all organoids exhibited either an increase in spike rate during exposure to light (presumed ON‐center RGCs; Figure 4A,B) or a decrease in spike rate (presumed OFF‐center RGCs; Figure 4C). The medians of the calculated change of firing (COF) were not significantly different between the methods for WT1 but they were for WT2 (Figure 4B, C). Indeed, WT2 organoids showed either a significant increase (Mann‐Whitney test: *P* = .009) or decrease (*P* = .042) in COF medians with Method I.

## 3 DISCUSSION

Generation of retinal organoids on a large scale is necessary in order to meet the growing demand for a model system which is predictive of human physiology and resembles key morphological and functional features. In this study, we used three human iPSC lines and differentiated them to retinal organoids using differentiation protocols activating either BMP4 (Method I) or IGF1 (Method II) signaling pathways; our data indicate that all cell lines were able to generate retinal cell types and the response to IGF1 and BMP4 was line‐ and method‐dependent. Variability in the propensity of iPSCs to differentiate is widely reported in the literature and is thought to be a result of some factors including gene expression heterogeneity among stem cell populations, DNA methylation and histone modifications, and differences in endogenous signaling activities, such as BMP4.^20^, ^21^, ^22^, ^23^ This is unsurprising, since BMP4 has been shown to be involved in the differentiation of the anterior portion of the neural plate toward retinal neurones; in addition, IGF1 promotes induction of retinal fate.^14^, ^24^ Interestingly, it has also been reported that using IGF1 in combination with a BMP4 antagonist results in efficient generation of cone photoreceptors.^25^ The interactions between these signaling pathways are complex, and it is plausible that depending on the endogenous levels of expression of key components of these pathways in the starting population of iPSCs it may be required to adapt differentiation protocols, which could include blocking BMP4 and simultaneously stimulating IGF1 in some cases.^23^ This is further compounded by the addition of other components (eg, T3, N2, RA) after day 18 of differentiation, which can synergize or agonize the activation of BMP4 or IGF1 pathways in a different way in each of the two methods reported in this article. Furthermore, this variability is also reflected in the ability to establish light‐sensitive signaling pathways. Only 7%‐12% of all RGCs changed the activity when light activates phototransduction in photorecetors and bipolar cells relay that signal to RGCs, whereas 2%‐5% of all RGCs were classified as potential ipRGCs. Except for WT2, there were no significant differences of light‐induced RGC activity between BMP4 and IGF1 protocols. High proportion of ipRGCs could be a reflection of maturity of the retinal organoids. In neonatal mice, functional ipRGCs can be observed at birth (P0), which is earlier than the establishment of light‐driven responses by RGCs (P10). Then, the number of ipRGCs decreases later in development.^26^

In this study we found that the two differentiation approaches produced bias toward certain retinal cell types, which was iPSC line‐ and method‐dependent. This shows that careful consideration is needed when choosing a differentiation protocol and that overall efficiency to generate retinal organoids would depend on the signaling pathways that are modulated.

## 4 CONCLUSION

Our data demonstrate that choice of cell line and differentiation protocol would depend on project requirements and further refinements in in vitro retinal culture methods are needed.

## CONFLICTS OF INTEREST

The authors indicated no potential conflicts of interest.

## AUTHOR CONTRIBUTIONS

V.C.: conception and design, collection and/or assembly of data, data analysis and interpretation, manuscript writing, final approval of manuscript; G.H.: collection and/or assembly of data, data analysis and interpretation, contributed to manuscript writing, final approval of manuscript; A.G.: data analysis and interpretation, final approval of manuscript; M.G.: collection and/or assembly of data, data analysis and interpretation, final approval of manuscript; M.C.: collection and/or assembly of data, final approval of manuscript; E.S.: conception and design, financial support, final approval of manuscript; M.L.: conception and design, financial support, collection and/or assembly of data, data analysis and interpretation, manuscript writing, final approval of manuscript; L.A.: conception and design, financial support, collection and/or assembly of data, data analysis and interpretation, manuscript writing, final approval of manuscript.

## DATA AVAILABILITY STATEMENT

The data that support the findings of this study are available in the supplementary material of this article.

## Supporting information


**Appendix S1**: Supplemental materials.

**Table S1** List of primary antibodies used for immunohistochemistry.
**Table S2**. List of oligonucleotides.

**Figure S1** Gene expression analysis for various retinal cell types at day 180 of differentiation. (A) Expression of *CRX* and *RHO* was significantly higher in WT1 organoids differentiated with Method II comparing to Method I; (B) WT2 differentiated with Method II resulted in significantly higher expression of *NRL* comparing to organoids differentiated with Method I; (C) Differentiating WT3 cells with Method I resulted in significant upregulation of all genes tested apart from *RBPMS* and *RPE65*, indicative of developing RGCs and RPE cells. Data is shown as mean ± SEM. At least 16 different organoids selected at random were used per sample. ****P < .0001 for all panels.
