# Supplementary material for: Human iPSC differentiation to retinal organoids in response to IGF1 and BMP4 activation is line‐ and method‐dependent
Source: Stem Cells. 2019 Dec 30;38(2):195–201. doi: 10.1002/stem.3116 (PMC7383896; doi:10.1002/stem.3116)
Supplement: Supplementary file 1 — Appendix S1: Supplemental materials. [file STEM-38-195-s001.docx]

**Supplemental materials**

***iPSC culture and retinal organoid differentiation***

iPSCs lines (WT1 [SB-AD2], WT2 [SB-AD3], WT3 [SB-AD4]) 1, 2 were cultured with mTeSR1 (Stem Cell Technologies) supplemented with penicillin/streptomycin (Thermo Fisher Scientific) on Matrigel Growth Factor Reduced (Corning)-coated plates at 37°C and 5% CO_2_ in a humidified incubator. Cell culture medium was replaced daily and cells were allowed to grow for 4-5 days prior to passaging at a ratio of 1:6.

Method I: iPSCs were grown until reaching 80-90% confluency, at which point they were dissociated with Accutase (Thermo Fisher Scientific) and seeded at a density of 7000 cells per well in lipidure (Amsbio)-coated 96 well plates in 100 μl mTeSR1 with 10 μM Y-27632 (Chemdea) (designated day -2) as described in our recent publication 3. On day 0 of differentiation medium was added, which comprised 41% IMDM, 41% Ham’s F12, 15% KOSR, GlutaMAX, penicillin/streptomycin, 1% chemically defined lipid concentrate (all Thermo Fisher Scientific), and 225 μM 1-Thioglycerol (Sigma), medium was changed every 2 days. At day 6, 2.25 nM BMP4 was added and medium was changed every 3 days. On day 18, medium was changed to DMEM/F12, GlutaMAX, penicillin/streptomycin, 2.5 μM SU5402 (Tocris), 1% N2 (Thermo Fisher Scientific) and medium was changed every 2 days. On day 24, medium was changed to DMEM/F12, GlutaMAX, penicillin/streptomycin, 10% FBS (Thermo Fisher Scientific), 1% N2, 0.5 μM retinoic acid (Sigma), 0.1 mM taurine (Sigma) and medium was changed 2-3 times a week thereafter.

Method II: This method was based on our previous recent publications 4-6; iPSCs were grown until reaching 80-90% confluency, at which point they were dissociated with Accutase (Thermo Fisher Scientific) and seeded at a density of 7000 cells per well in lipidure (Amsbio)-coated 96 well plates in 100 μl mTeSR1 with 10 μM Y-27632 (Chemdea) (designated day -2). The base of the differentiation medium comprised DMEM/F12, MEM NEAA (Thermo Fisher Scientific), 1% B27 (Thermo Fisher Scientific), and penicillin/streptomycin. On days 0-4, medium was supplemented with 20% KOSR and 5 ng/ml IGF1 (Sigma). On days 5-8, medium was supplemented with 15% KOSR and 5 ng/ml IGF1. On days 9-18, medium was supplemented with 10% KOSR and 5 ng/ml IGF1. On days 18-34, medium was supplemented with 10% FBS, 5 ng/ml IGF1, 0.5 μM retinoic acid, 0.1 mM taurine, and 40 ng/ml T3 (Sigma). From day 35 onwards, medium was supplemented with 10% FBS, 10 ng/ml IGF1, 0.5 μM retinoic acid, 0.1 mM taurine, 40 ng/ml T3, and 1% N2 4-6.

***Immunofluorescence***

Organoids were washed with PBS and fixed in 4% paraformaldehyde for 20 minutes followed by several washing steps with PBS. They were subsequently dehydrated in 30% sucrose at 4°C overnight, embedded in Optimal Cutting Temperature (OCT) embedding matrix (Cell Path Ltd) and cryosectioned. For immunofluorescence, sections were rinsed in PBS and incubated for 1 hour in blocking buffer (5% normal goat serum and 0.3% Triton-X-100 in PBS) at room temperature. All antibodies (**Supplementary Table 1**) were diluted in 1% bovine serum albumin and 0.3% Triton-X-100 in PBS. Primary antibodies were applied overnight at 4°C. Secondary antibodies conjugated to Alexa488 (Life Technologies) or Cy3 (Jackson Immuno Research Laboratories) were applied for 1 hour at room temperature. Sections were washed with PBS and mounted with Vectashield (Vector Laboratories) containing Hoechst 33042 (Life Technologies). For quantification analysis, between 5 and 8 organoids were used.

***Quantitative polymerase chain reaction (qPCR)***

RNA was isolated using ReliaPrep™ RNA Cell Miniprep System (Promega) following the instructions of the manufacturer. cDNA was synthesised using GoScript™ Reverse Transcription System (Promega) according to the manufacturer’s instructions. qPCR was performed in triplicate reactions using GoTaq® qPCR Master Mix reagent system (Promega). The reaction was run on the Applied Biosystems® QuantStudio™ 7 Flex Real-Time PCR System (Life Technologies). The data were analysed using the QuantStudio™ software (Life Technologies) and relative gene expression was determined using the 2^-ΔΔCt^ method using *GAPDH* as a housekeeping gene and adult human retina cDNA as a reference sample. The list of oligonucleotides is shown in **Supplementary** **Table 2**.

***Electrophysiology***

Electrophysiology experiments were carried out as described in Hallam et al., 2018 3. Briefly, organoids were transferred to 34°C artificial cerebrospinal fluid (aCSF) containing the following (in mM): 118 NaCl, 25 NaHCO3, 1 NaH2 PO4, 3 KCl, 1 MgCl2, 2 CaCl2, 10 glucose, 0.5 l-Glutamine and 0.01 9-cis-retinal. Organoids were opened longitudinally and placed, with the presumed retinal ganglion cell (RGC) layer facing down onto the 4096 channel multielectrode array. Recordings were performed on the BioCam4096 MEA platform with BioChips 4096S+ (3Brain GmbH, Lanquart, Switzerland).

Broad white (high photopic) light pulses (WLP, 200 ms, 217 µW/cm^2^ irradiance, 1Hz) were flashed for 5 minutes onto the organoids following recording of spontaneous activity in the dark for 5 minutes. To single out the sustained intrinsically photosensitive RGCs (ipRGCs) responses, we also used sustained broad blue light stimulation (same irradiance as WLP) and discriminated the transient photoreceptor-driven responses from the stimulus onset.

RGCs were considered responsive if they showed at least 25% increase or decrease in spiking activity during 30 seconds after WLP onset compared to a similar time window before the light was turned on. For each cell, all spikes occurring during these two time windows were counted and the mean % change (± SEM) in activity between windows was calculated. A total of 5-6 organoids were recorded for each condition. Statistical significance (Wilcoxon rank sum test) and firing rate analyses were evaluated by using MATLAB (Mathworks, MA).

**References**

1. Buskin A, Zhu L, Chichagova V, et al. Disrupted alternative splicing for genes implicated in splicing and ciliogenesis causes PRPF31 retinitis pigmentosa. Nat Commun*.* 2018;9:4234.

2. Melguizo-Sanchis D, Xu Y, Taheem D, et al. iPSC modeling of severe aplastic anemia reveals impaired differentiation and telomere shortening in blood progenitors. Cell death & disease*.* 2018;9:128.

3. Hallam D, Hilgen G, Dorgau B, et al. Human-Induced Pluripotent Stem Cells Generate Light Responsive Retinal Organoids with Variable and Nutrient-Dependent Efficiency. Stem Cells*.* 2018;36:1535-1551.

4. Dorgau B, Felemban M, Sharpe A, et al. Laminin gamma3 plays an important role in retinal lamination, photoreceptor organisation and ganglion cell differentiation. Cell death & disease*.* 2018;9:615.

5. Felemban M, Dorgau B, Hunt NC, et al. Extracellular matrix component expression in human pluripotent stem cell-derived retinal organoids recapitulates retinogenesis in vivo and reveals an important role for IMPG1 and CD44 in the development of photoreceptors and interphotoreceptor matrix. Acta Biomater*.* 2018;74:207-221.

6. Dorgau B, Felemban M, Hilgen G, et al. Decellularised extracellular matrix-derived peptides from neural retina and retinal pigment epithelium enhance the expression of synaptic markers and light responsiveness of human pluripotent stem cell derived retinal organoids. Biomaterials*.* 2019;199:63-75.
