# Supplementary material for: Human iPSC differentiation to retinal organoids in response to IGF1 and BMP4 activation is line‐ and method‐dependent
Source: Stem Cells. 2019 Dec 30;38(2):195–201. doi: 10.1002/stem.3116 (PMC7383896; doi:10.1002/stem.3116)
Supplement: Supplementary file 2 — Table S1 List of primary antibodies used for immunohistochemistry. Table S2. List of oligonucleotides. [file STEM-38-195-s002.docx]

**Supplementary Table 1. List of primary antibodies used for immunohistochemistry.**

| **Antibody** | **Host** | **Dilution** | **Supplier** |
| --- | --- | --- | --- |
| RECOVERIN | Rabbit | 1:1000 | Millipore, ab5585 |
| RHODOPSIN (RET-P1) | Mouse | 1:200 | Sigma-Aldrich, O4886 |
| OPSIN RED/GREEN | Rabbit | 1:200 | Millipore, ab5405 |
| OPSIN BLUE | Rabbit | 1:200 | Millipore, ab5407 |
| AP-2α | Mouse | 1:100 | Santa Cruz Biotechnology Inc., sc-12726 |
| Synuclein gamma (SNCG) | Mouse | 1:500 | Abnova, H00006623-M01A |
| CRALBP | Mouse | 1:100 | GeneTex, GTX15051 |
| PROX1 | Rabbit | 1:1000 | Millipore, ab5475 |
| PKCα | Mouse | 1:200 | BD Transduction laboratories, 610107 |

**Supplementary Table 2. List of oligonucleotides.**

| **Gene** | **Forward primer** | **Reverse primer** |
| --- | --- | --- |
| *CRX* | GTGAGGAGGTGGCTCTGAAG | CTGCTGTTTCTGCTGCTGTC |
| *RCVRN* | TTCAAGGAGTACGTCATCGCC | GATGGTCCCGTTACCGTCC |
| *NRL* | GGGCTGAGTCCTGAAGAGG | TTTAGCTCCCGCACAGACAT |
| *RHO* | GGTCCAAAGACACCTGATGG | TGTTGGCAACCCACTAATGA |
| *OPN1SW* | ATACCGCAGCGAGTCCTATAC | GATCCTACCATCACAACCAC |
| *OPN1MW* | CATCTTTGGTTGGAGCAGGTACT | TCTCTGCCTTCTGGGTGGAT |
| *OPN1LW* | GCCTACTTTGCCAAAAGTGC | GATGAGACCTCCGTTTTGGA |
| *PROX1* | TGACTTTGAGGTTCCAGAGAGA | CTCTTGTAGGCAGTTCGGGG |
| *RLBP* | GGCAGGGAACAACCAAGACT | AGTCAGGGCCAAGTTGTGAC |
| *AP2a* | GTTACCCTGCTCACATCACTAG | TCTTGTCACTTGCTCATTGGG |
| *MATH5* | CCCTAAATTTGGGCAAGTGAAGA | CAAAGCAACTCACGTGCAATC |
| *RBPMS* | TGACAGTCGCTCAGAAGCAG | TCACACACCTGGGACATAGT |
| *RPE65* | GCCCAGGAGCAGGACAAAAG | GCGCATCTGCAAGTTAAAACCA |
| *GAPDH* | TGCACCACCAACTGCTTAGC | GGCATGGACTGTGGTCATGAG |
