# Supplementary figures and images for: Human iPSC differentiation to retinal organoids in response to IGF1 and BMP4 activation is line‐ and method‐dependent
Source: Stem Cells. 2019 Dec 30;38(2):195–201. doi: 10.1002/stem.3116 (PMC7383896; doi:10.1002/stem.3116)

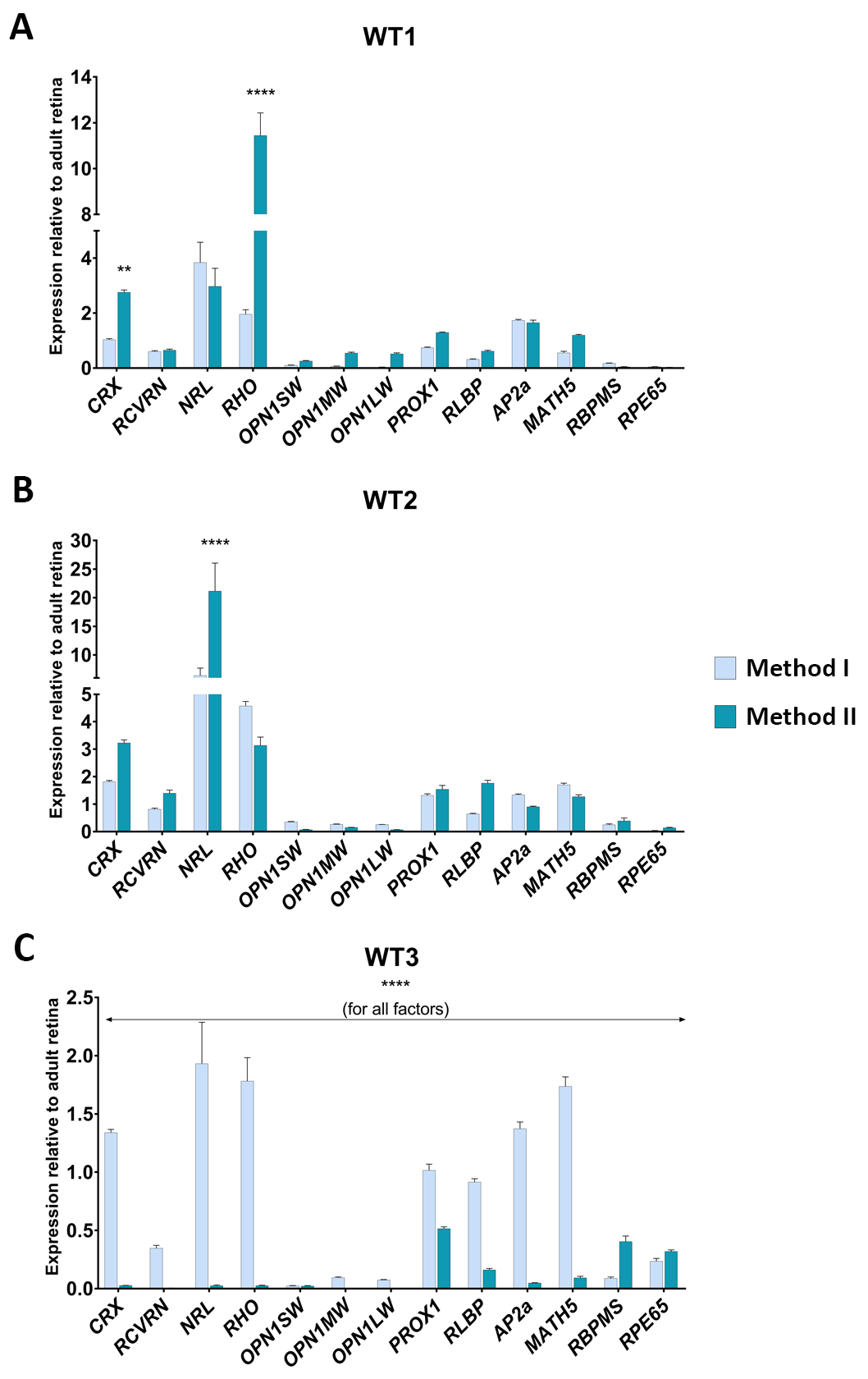

Supplement: Supplementary file 3 — Figure S1 Gene expression analysis for various retinal cell types at day 180 of differentiation. (A) Expression of CRX and RHO was significantly higher in WT1 organoids differentiated with Method II comparing to Method I; (B) WT2 differentiated with Method II resulted in significantly higher expression of NRL comparing to organoids differentiated with Method I; (C) Differentiating WT3 cells with Method I resulted in significant upregulation of all genes tested apart from RBPMS and RPE65, indicative of developing RGCs and RPE cells. Data is shown as mean ± SEM. At least 16 different organoids selected at random were used per sample. ****P < .0001 for all panels. [file STEM-38-195-s003.tif]
